# A Study on the Detection of Internal Defect Types for Duct Depth of Prestressed Concrete Structures Using Electromagnetic and Elastic Waves

**DOI:** 10.3390/ma14143931

**Published:** 2021-07-14

**Authors:** Young-Geun Yoon, Jae-Yun Lee, Hajin Choi, Tae-Keun Oh

**Affiliations:** 1 Department of Safety Engineering, Incheon National University, Incheon 22012, Korea; yyg900@inu.ac.kr (Y.-G.Y.); jaeyun.lee@daewooenc.com (J.-Y.L.); 2 School of Architecture, Soongsil University, Seoul 06978, Korea; 3 Research Institute for Engineering and Technology, Incheon National University, Incheon 22012, Korea

**Keywords:** prestressed concrete, electromagnetic wave, elastic wave, non-destructive testing, impact echo, principal component analysis

## Abstract

Prestressed concrete (PSC) is widely used for the construction of bridges. The collapse of several bridges with PSC has been reported, and insufficient grout and tendon corrosion were found inside the ducts of these bridges. Therefore, non-destructive testing (NDT) technology is important for identifying defects inside ducts in PSC structures. Electromagnetic (EM) waves have limited detection of internal defects in ducts due to strong reflections from the surface of the steel ducts. Spectral analysis of the existing impact echo (IE) method is limited to specific conditions. Moreover, the flexural mode in upper defects of ducts located at a shallow depth and delamination defects inside ducts are not considered. In this study, the applicability of the elastic wave of IE was analyzed, and multichannel analysis of surface, EM, and shear waves was employed to evaluate six types of PSC structures. A procedure using EM waves, IE, and principal component analysis (PCA) was proposed for a more accurate classification of defect types inside ducts. The proposed procedure was effective in classifying upper, internal, and delamination defects of ducts under 100 mm in thickness, and it could be utilized up to 200 mm in the case of duct defect limitations.

## 1. Introduction

Prestressed concrete (PSC) is widely used in modern bridge construction. During the construction process, grouting is required to reduce the corrosion of tendons in ducts located inside the PSC structure. However, limitations of grouting technology and improper handling often result in grout defects inside the tendon ducts. For example, insufficient grout and induced corrosion of tendons were found in the duct of Malle Bridge, a 20-year-old bridge in Flemish Belgium, after its collapse [1,2]. In addition, severely corroded tendons in these structures have been reported [3]. As the reliability and durability of these PSC structures deteriorate due to corroded steel, structural failure or collapse may occur, as in the case of the Malle Bridge and Ynys-Y-Gwas Bridge [4,5]. Several European researchers attached load cells and anemometers to the entire bridge and conducted monitoring studies on wind speed and load according to actual vehicle movement and environment. Although these studies can confirm the load concentration of the bridge, there is a limit in identifying internal defects [6]. Therefore, it is necessary to evaluate the condition of bridges through the application of non-destructive testing (NDT) to inspect the structure without causing damages. NDT methods widely used to evaluate the condition of concrete include electromagnetic (EM) waves, ultrasonic waves, and impact echoes (IEs). Traditional EM wave methods have limitations in detecting grout defects in tendon ducts because tendons and metal ducts can shield electrical and magnetic signals [7,8], but strong reflections on steel ducts and tendons can be an advantage when combined with other NDT methods. The synthetic aperture focusing technique (SAFT) method for shear waves (S waves), which has high energy among ultrasonic waves, was effective in imaging delamination defects, voids, and reinforcing bars inside concrete, and sensitively responded to tendons inside ducts of PSC structures [9,10]. However, it may be difficult to distinguish between voids and tendons caused by insufficient grouting of the duct. In this study, the feasibility of the IE method for non-destructive detection of various types of tendon duct defects that may occur is evaluated, and an optimal procedure is proposed through the combination of NDT methods such as EM waves, S waves, and IE to classify defects by type.

The IE method introduced by Sansalone et al. in the 1980s enabled to detect defects in structures [11,12]. In this method, a wave propagation signal is obtained in the time domain using a mechanical pulse generated by a physical impact. The displacement signal is measured by an accelerometer placed adjacent to the impactor on the surface. The time-domain signal is transformed into a frequency domain spectrum, and given the characteristic frequency obtained from the spectrum and the wave velocity of the slab, the thickness of an intact slab can be calculated. In addition to measuring the thickness of slabs, the IE method has been continuously applied to examine defects [11], cracks [13], voids and debonding [14], honeycomb [15], delamination, and interface quality [16,17]. In addition, condition assessments of many concrete structures have been performed, such as bridge deck inspection [18], bridge deck crack detection [19], internal defect assessment of post-tension concrete slabs in high-rise buildings [20], rebar corrosion damage detection [21], and shotcrete adhesion condition evaluation [22]. The IE method is non-destructive, non-invasive method that is suitable for cross-section detection, is easy to use, and has high applicability in terms of deep detection [23,24,25]. A multichannel analysis of surface waves (MASW) is considered an advance of the IE method with various advantages. The MASW contributed to various evaluations of reinforced concrete (RC) slabs by measuring the R wave velocity of concrete via the convergence of the A0 and S0 modes based on theoretical curves and by analyzing various higher order modes in addition to the thickness mode (S1) [26,27,28]. As the MASW generally assumes an infinite reputation, no study has been conducted on PSC structures; thus, it is necessary to review its applicability.

In addition to the detection of voids and delamination defects in RC slabs, the IE method has been applied to the detection of duct defects in PSC structures [29,30,31,32]. For empty ducts, ducts with insufficient grouting, and deep ducts, defects were detected through an experimental approach to the reflected wave in the duct according to the shift of the spectral thickness mode frequency and the degree of grouting. However, several variables have not been studied in detail, such as the flexural mode according to the degree of grouting and delamination defects inside the duct due to continuous load. These variables are important in the reliability and efficiency of the IE method because they are commonly present in actual PSC structures. Previous studies have shown that the depth and internal condition of ducts can affect the frequency, but the extent of this change is not clear. Recently, to evaluate the duct condition of PSC structures, some studies have been conducted to predict the types of defects that are significantly different by applying machine learning (ML) to the signal of IE and additional information (concrete thickness and duct depth) [33,34]. However, a model without analysis of the IE spectrum is simply a black box-type ML model, and the misclassification rate increases when it deviates from the trained situation. It is necessary to consider an influential variable for the classification of defect types through elaborate spectrum analysis and feature extraction rather than directly applying ML to the IE signal.

This study resolves this gap by systematically studying the effects of the PSC duct on EM waves, S waves, and IE spectra. In addition, the flexural mode of ducts with deficient grouting installed at shallow depths and delamination defects inside ducts, which were not considered in previous studies, are considered. Principal component analysis (PCA) is performed to provide insights into the phenomena observed according to the type of defect by analyzing the overall spectral characteristics, which consider the frequency and the changed frequency exhibited by various reflected waves.

## 2. Materials and Methods

### 2.1. Materials and Preparation of Specimens

**Figure 1:**
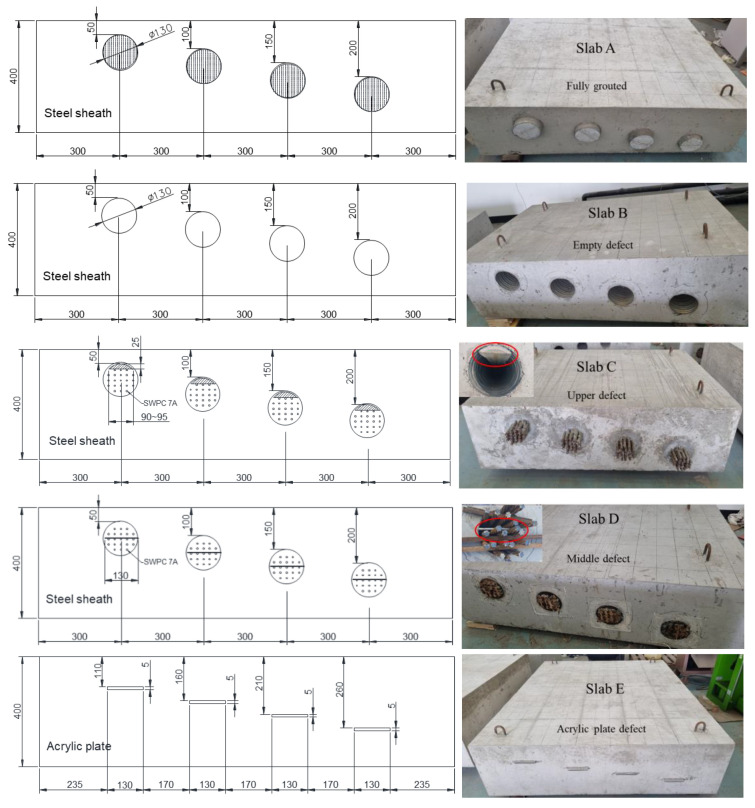
Cross-sectional dimensions (in mm) and manufacturing results of the tested concrete specimens.

Several concrete specimens containing various defects were constructed according to standard concrete casting and curing criteria. The thickness of the test piece was 400 mm, and defects were installed using a steel duct and acrylic plate. The drawings and manufacturing results are shown in Figure 1. Slab A had a fully filled duct, slab B had an empty duct, slab C had an incompletely filled duct on top with insufficient grouting, slab D had a duct with a delamination defect in the middle of the duct, and slab E had a duct with a general delamination defect. For slabs A to D, four duct depths of 50, 100, 150, and 200 mm were examined, and for slabs D to E, delamination defects with four depths of 110, 160, 210, and 260 mm were considered. Testing for a depth of 400 mm (N400) of intact concrete without ducts and defects was conducted in the area between ducts and between acrylic plates. 

### 2.2. Electromagnetic Wave Measurement with RC Radar

**Figure 2:**
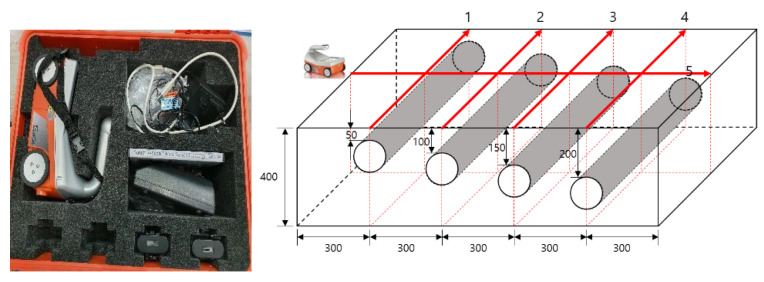
EM wave measuring equipment and test procedures (dimensions in mm).

EM waves are reflected from the interface of two objects with different permittivity. Based on this basic principle, RC radar for concrete is extensively used, and it easily verifies poor construction or internal reinforcement information because it responds sensitively to reinforcing bars, piping, and PC steel inside concrete. In this study, the StructureScan Mini LT (GSSI) was used as the RC radar equipment. This equipment has a measurement depth of 500 mm and was suitable for the specimens in this study. As shown in Figure 2, the test was conducted at four duct lines and one line in the direction perpendicular to the duct. Five specimens were measured at the same point.

### 2.3. S Wave Measurement with Pundit 250 Array

**Figure 3:**
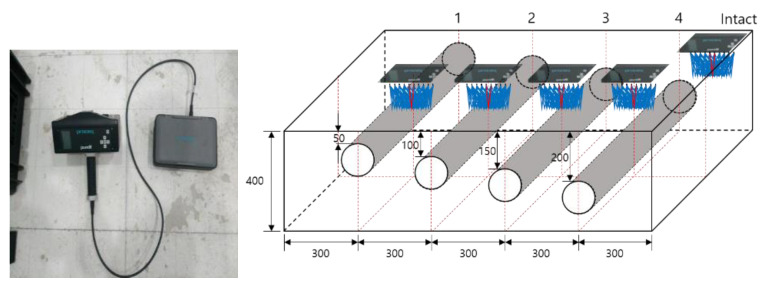
Shear wave measuring equipment and test procedure (dimensions in mm).

A tomography (B scan) analysis using S wave was performed using a Pundit 250 array with 24 sensors, as shown in Figure 3. Array equipment with dry point contact technology collects 56 A-scans at one point of measurement, and real-time B scans are obtained using the SAFT algorithm. The S wave was measured at four depths and at intact positions, and it was measured at the same point for a total of five specimens.

### 2.4. Elastic Wave Measurement with IE and MASW

**Figure 4:**
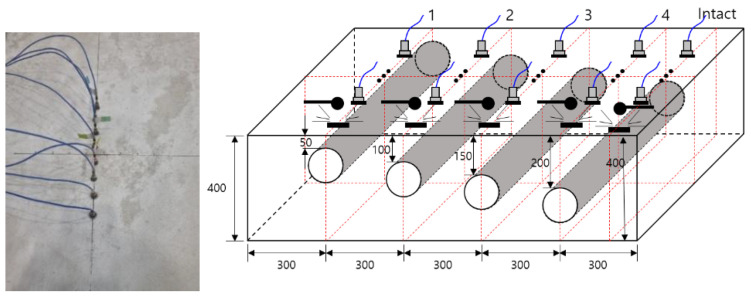
Test setup and procedure for measuring elastic waves with IE test (dimensions in mm).

To effectively measure the dynamic response, a multi-sensor was attached to the concrete surface, as shown in Figure 4, and an elastic wave was generated and measured. The use of multiple sensors minimizes noise and enables one to achieve a high signal-to-noise ratio and good resolution. In this study, a steel ball hammer with a diameter of 20 mm was used to generate elastic waves on a concrete specimen. This hammer is suitable for generating very low to 15 kHz frequency signals. To measure the dynamic response of the concrete specimen, an accelerometer (PCB 353B16, PCB, NY, USA) with a resonance frequency of approximately 70 kHz and an error of 5% in the range of 10 kHz or less was used. The IE test was performed by placing eight accelerometers with the same specifications at intervals of 30 mm, and the signal measured by the accelerometer was stabilized by a signal conditioner (PCB 482C16, PCB, NY, USA) and digitized with a sampling frequency of 1 MHz using an oscilloscope (NI-PXIe 6366). The time signal was transformed into the frequency domain using a fast Fourier transform (FFT) algorithm. The frequency spectrum and phase velocity dispersion curve obtained by MASW for eight accelerometers were analyzed.

**Figure 5:**
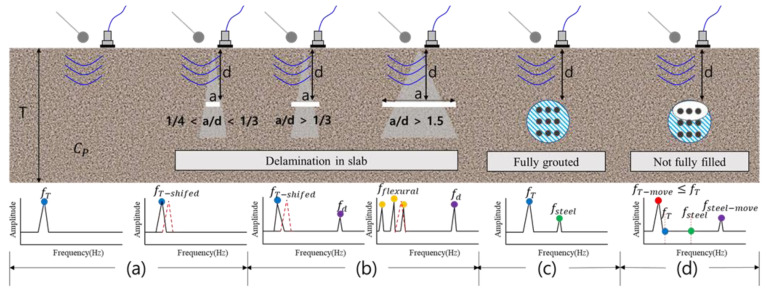
Summary of changes of IE spectra by type of defect identified in previous studies.

IE has been studied for various types of defects, and its characteristics are shown in Figure 5. In Figure 5a, the estimation of the thickness mode frequency (*ft*) for concrete without internal defects is the same as in Equation (1) [11] (p. 2). If there are voids and cracks with 1/4 < *a/d* < 1/3 inside, *ft* is shifted to a lower frequency. Here, β is the correction factor, $C_{P}$ is the *p*-wave speed, and T is the slab thickness.

(1) $$f_{T}=\frac{\beta C_{P}}{2T}$$

In Figure 5b, when *a/d* is higher than 1/3, *ft* is shifted, and the thickness of the defect (*fd*) can be detected, which is calculated using Equation (2) [12] (p. 2). It has been reported that when *a/d* exceeds 1.5, the amplitude of *fd* increases and a strong flexural mode occurs [35]. The formula used to calculate the flexural mode varies depending on the structurally fixed shape, and the fixed-fixed state was considered in this study. *d* is the distance between the duct or the defect and the surface.

(2) $$f_{T}=\frac{\beta C_{P}}{2d}$$

The IE for the duct was studied for the conditions of Figure 5c,e and the hollow case. In the fully grouted state (Figure 5c), *ft* is the same as in Figure 5a, and *fsteel* occurs owing to the reflected wave in the steel duct. The calculation method for this is the same as in Equation (3) [12] (p. 2). It has been reported that the correction factor *β* is not used in Equation (3), and *fsteel* may be shifted owing to various experimental influences.

(3) $$f_{steel}\approx \frac{C_{P}}{4d}$$

Figure 5d represents a defect type that can occur because of insufficient grouting or unfilled top [12] (p. 2). In this case, shifts in *ft* and *fsteel* may occur because of the increase in the movement distance of the elastic wave, and the main frequency is estimated through Equations (4)–(6).

(4) $$T_{move}\geq T,\;f_{steel-move}\approx 2\times f_{steel}$$

(5) $$f_{T-move}=\frac{\beta C_{P}}{2T_{move}}$$

(6) $$f_{steel-move}=\frac{\beta C_{P}}{2d}$$

In previous studies, the internal states of RC and PSC were determined through analysis of the basic IE equation according to Equations (1) and (6). However, when using an accelerometer for the IE test, experimental errors may occur depending on the duct, tendon, depth, etc., and the degree of shifting frequency due to the influence of the duct and defects is not clear; therefore, additional interpretation of the measurement results is required. Even if accurate measurement is possible, hammers with different diameters depending on the depth must be selectively used, and high-frequency generation for detecting ducts and defects disposed close to the surface is limited. Therefore, in this study, eight accelerometers were simultaneously used to systematically overcome the limitations of existing studies, and analysis was performed by detecting the flexural mode (*ff*) rather than the high-frequency generation using only a 20 mm hammer for excitation.

## 3. Feature Extraction Methods

### Principal Component Analysis

**Figure 6:**
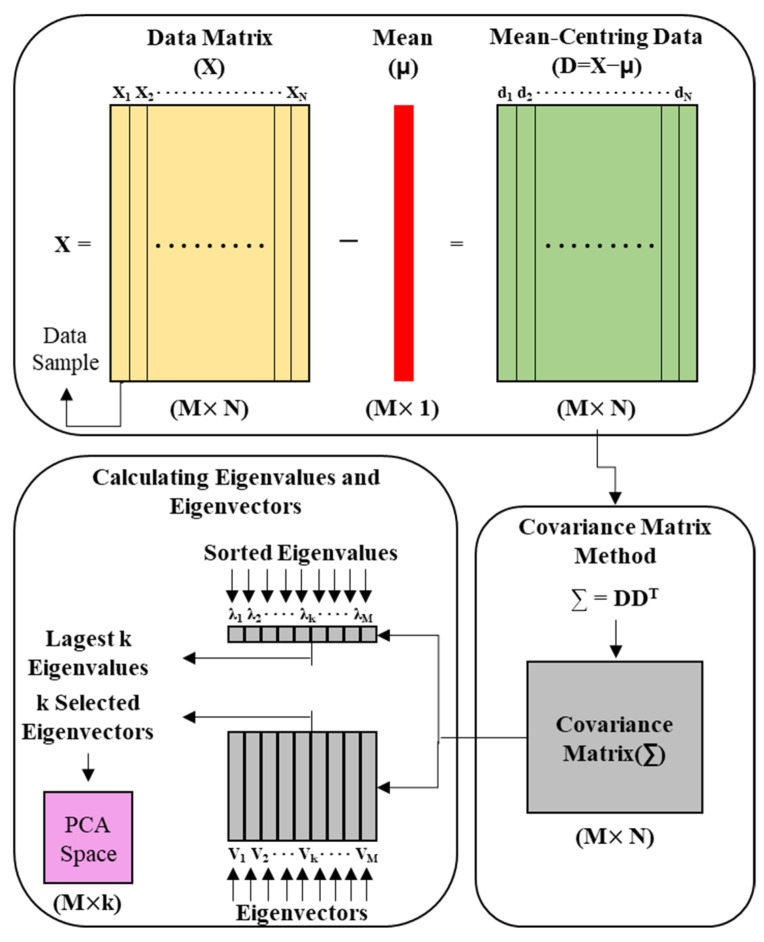
Procedure of PCA.

PCA uses orthogonal transformations to transform samples in a high-dimensional space that are likely to be related to each other into a low-dimensional space. PCA was proposed by Pearson (1901) and later developed by Hotelling (1936) and Jolliffe to establish the modern theory [36,37]. PCA linearly transforms data into a new coordinate system such that when data are mapped to one axis, the axis with the largest variance is placed as the first principal component and the second largest component is placed as the second principal component. Therefore, PCA is a method of dividing the sample difference into components that best represent such difference, and the procedure is shown in Figure 6 [38]. Because it is not possible to quantify the degree to which the major frequency that theoretically occurs in the IE spectrum is shifted by type, PCA was used in this study to derive the main components for *ft*, *ff*, and a certain range of features appearing in the IE spectrum for the duct and delamination defects.

## 4. Results and Discussion

### 4.1. Experimental Result for RC Radar

**Figure 7:**
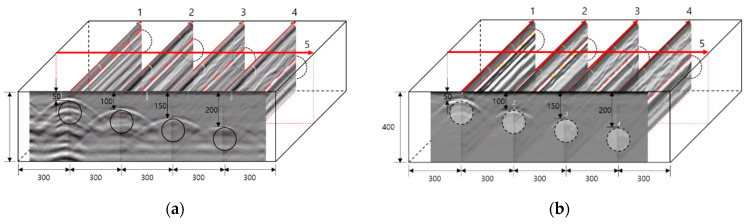

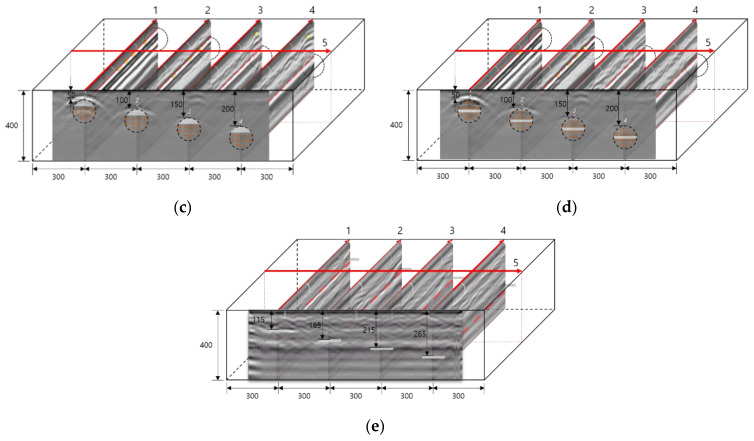
Test results by RC radar. Slabs: (**a**) A; (**b**) B; (**c**) C; (**d**) D; (**e**) E (dimensions in mm).

Tests were performed following the same procedure for specimens A to E using RC radar, and the resulting images were extracted using RADAN7, a commercial program provided by GSSI. The results of matching the extracted B scan to five specimens are shown in Figure 7. In general, when the duct was close to the concrete surface, the energy of the reflected wave was large, and it weakened when the depth of the duct was increased. In the B scan, most of the energy was reflected from the steel duct; thus, it was difficult to evaluate the degree of grouting inside the concrete, and the delamination defect depicted with the acrylic plate in Figure 7e was not detected. The permittivity of concrete ranges from 5 to 12, the permittivity of steel is 200 or more, and reflected waves are strongly generated due to this large permittivity difference. However, because acrylic has a permittivity of 2.56, the difference is smaller than that of air (permittivity 1) due to delamination defects, which hinders the detection of defects.

**Figure 8:**
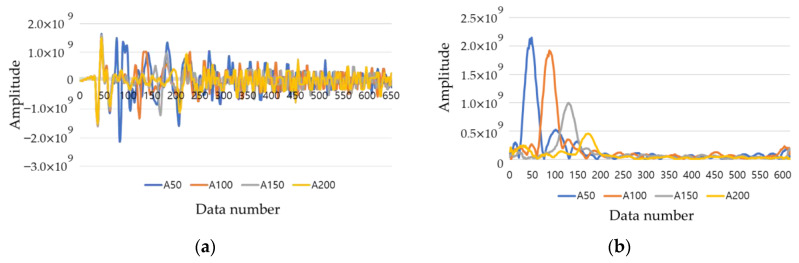
Raw data analysis for EM wave. (**a**) Raw data; (**b**) Hilbert transform result.

**Figure 9:**
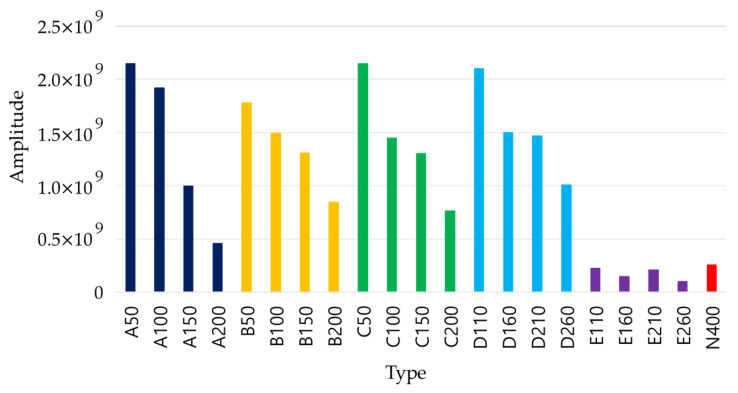
Comparison of attenuation for reflected waves of EM waves by type.

As shown in Figure 7, when commercial equipment is used, a quantitative analysis is difficult because only a rough evaluation on the extracted B scan is possible. Therefore, the analysis was conducted using raw data collected from RC radar. Figure 8a shows the raw data at the center of the duct of slab A, and Figure 8b shows the result of performing the Hilbert transform. Through the converted result, it is possible to determine the degree of energy attenuation according to the location and depth of the duct. The same procedure was performed for slabs A to E at the normal position, and the results are shown in Figure 9.

Compared with slab E and N400, the existence of ducts in slabs A to D was sufficiently detectable up to a depth of 200 mm. In the case of slabs B to D with defects inside the duct, the difference was insignificant, and it was difficult to distinguish the types of defects. Because the depth of the duct can be accurately identified and the input energy is the same, the presence of internal defects in the duct at a specific depth can be identified by comparing the energy attenuation of the main peak.

### 4.2. Experimental Result for Pundit 250 Array

**Figure 10:**
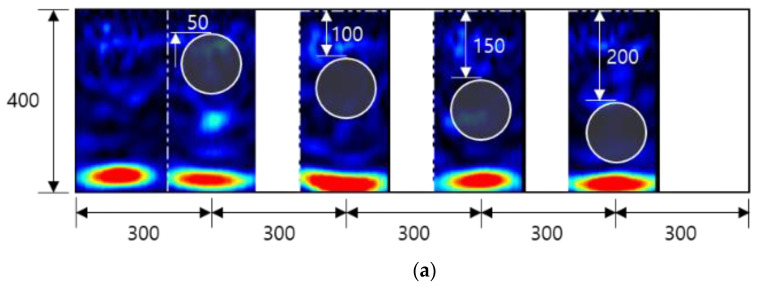

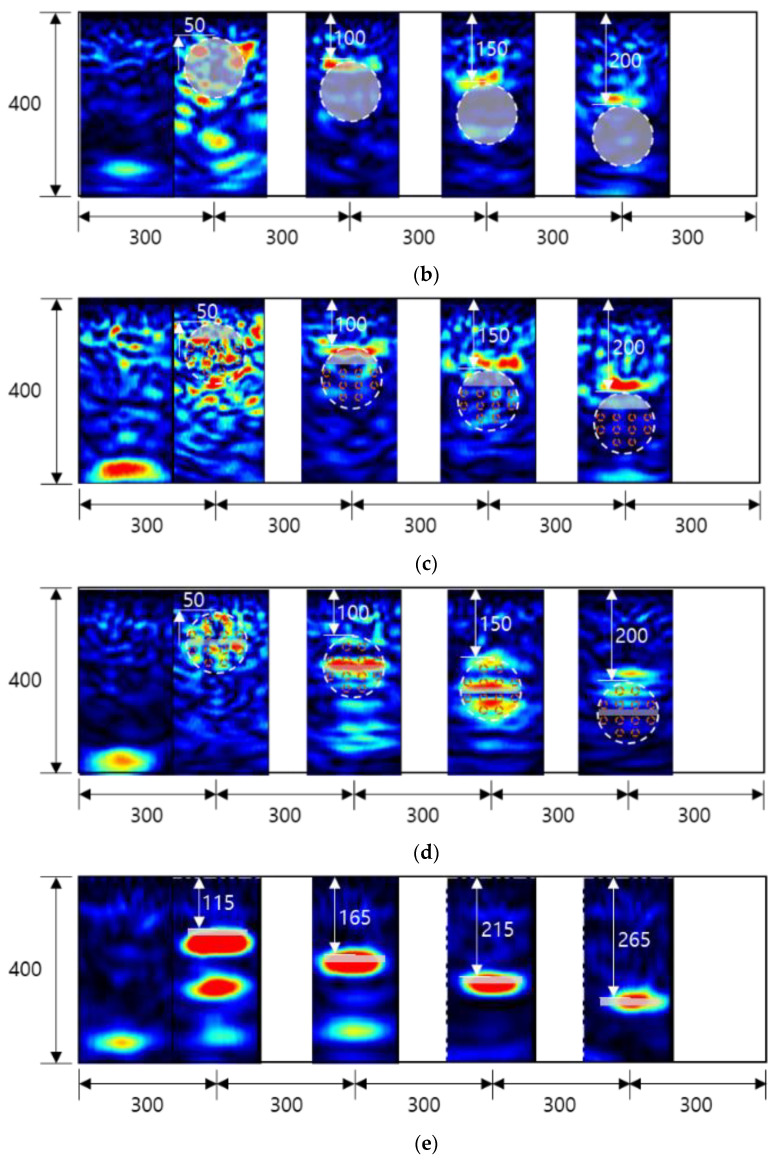
B scan results by Pundit 250 array. Slabs: (**a**) A; (**b**) B; (**c**) C; (**d**) D; (**e**) E (dimensions in mm).

Figure 10 shows the results of the B scan extracted using PL-Link, a program provided by Proceq, after performing the test in the same manner for slabs A to E using the Pundit 250 array. The Pundit 250 array is effective in detecting voids, reinforcing bars, and defects in concrete using S waves at 50 kHz [9,10] (p. 2). In this study, its applicability to PSC structures was considered.

In slab A, which has a fully grouted duct, the duct was not detected, and only the thickness of the slab was detected. It seems that the thickness of the duct (approximately 1 mm) is thin compared to the wavelength of the S wave, such that it cannot be detected in this case. In the case of slab B, B50 was scattered owing to energy dissipation on the surface, and in the cases of B100, B150, and B200, the position of the upper part of the duct was roughly identified. Slab C showed a similar tendency as slab B, and it was difficult to distinguish between empty and upper defects with the Pundit 250 array. In the case of D50 of slab D, which had a delamination defect inside the duct, the reflected wave of the tendon inside the duct was identified, the intermediate defect of D100–200 showed a large reflected wave, and some of the waves reflected by the tendon were detected. In the delamination defect of slab E represented with an acrylic plate, the reflected wave clearly appeared regardless of the depth.

In the analysis using the Pundit 250 array, the duct without tendon arrangement was difficult to detect. Therefore, when the tendon was placed in the actual structure, the reflected wave of the tendon, the void, and the delamination defect in the duct appeared with the same intensity, being difficult to distinguish.

### 4.3. Experimental Result for MASW

**Figure 11:**
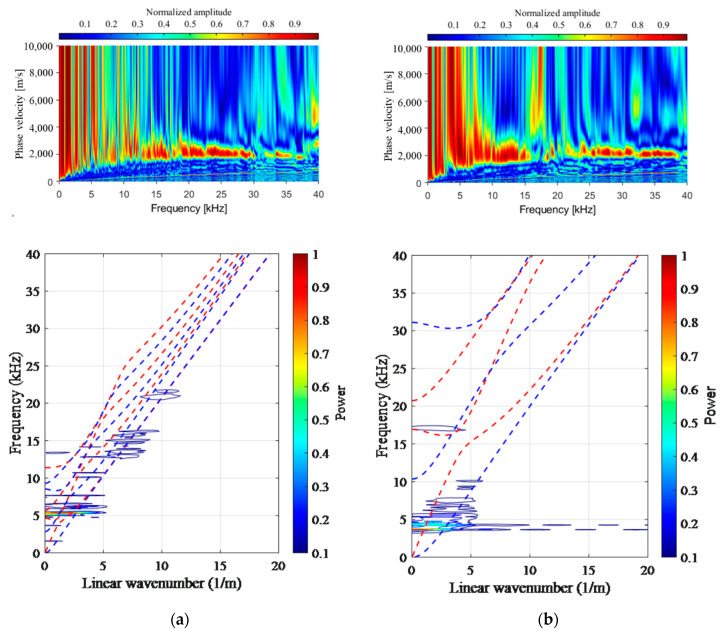
Phase velocity dispersion curves for normal and delamination defects. (**a**) Intact 400 mm (N400); (**b**) acrylic plate defect 110 mm (E110).

To analyze the impact response, the applicability of the MASW using eight sensors was first reviewed. Figure 11 shows two types of variance curves used in the MASW analysis. N400 generally followed the A0 mode, and the theoretical S1 (thickness) mode showed a strong frequency at 5500 Hz. E110 was able to measure the thickness of the delamination defect through the S1 mode of the floor, but it was confirmed that the floor thickness mode was shifted to a low frequency. This is attributed to the increase in the movement distance of the elastic wave due to the delamination defect. In the case of slabs A–D, the theoretical curve was not followed, and as the movement distance of the elastic wave due to the duct increased, only the shifting of the thickness mode for the floor to a low frequency below 5 kHz was confirmed. As a result, some A0 and S1 modes could be confirmed through MASW analysis. However, because the shape of the duct was different from the plate-shaped defect and was also affected by the duct, it was difficult to quantify and identify the type of defect. In addition, the theoretical S1 mode at 35–40 kHz for B50 and C50 did not appear, and a strong frequency occurred between 4 and 9 kHz. The reason for this result is that a flexural mode appearing in the delamination defect with *a/d* higher than 1.5 was strongly generated. Therefore, the MASW analysis is effective under the premise of an infinite plate, and it seems that a different approach is needed to identify defects in ducts in PSC structures.

### 4.4. Experimental Result for IE

**Figure 12:**
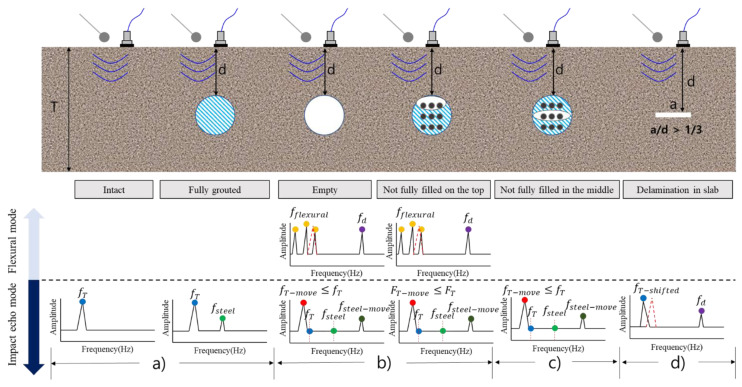
Six types tested and expected spectra in this study.

Previous studies on the occurrence of the IE and flexural modes have been conducted for plate-shaped (delamination) defects. However, in the PSC structure, the detection based on the IE mode has been performed without considering the flexural mode for the defect in the upper part of the duct, which may be difficult to identify due to the limitation of high-frequency generation and the error of the experiment. In this study, as shown in Figure 12, the flexural mode at the top of the duct and intermediate delamination defects that could be caused by tendon corrosion were also considered. In addition, through multi-sensor analysis, the noise, which is a limit point when measuring with one existing sensor, was supplemented, and the reliability of the analysis was improved.

The theoretical approach of the flexural mode due to the shape and boundary conditions of the duct is difficult. Therefore, in this study, considering the shape at the top of the duct, a method of calculating the natural frequency for two beams and assuming a range was applied [35]. First, *a/d* was calculated to confirm the occurrence of flexural mode by type, and the floor thickness mode (*ft*), flexural mode (*ff*), and mode for duct or acrylic plate (*fd*) based on the results of previous studies are summarized in Table 1. B50 and C50 are expected to have a strong *ff* with *a/d* of 1.5 or more; in the case of B100, some weak *ff* may be included. Otherwise, because the IE mode is dominant, *ff* does not occur, and the peaks of *ft* and *fd* may be shifted.

The concrete at the top of the duct is fixed, and the angular frequency ($\omega_{n}$) for the natural frequency is calculated using Equation (7) [39]:

(7) $$\omega_{n}=C_{n}\sqrt{\frac{E_{d}I}{\bar{m}L^{4}}}$$

Here, $C_{n}$ is a constant (22.3733) for the first-order natural frequency, $E_{d}$ is the dynamic modulus of elasticity, I is the moment, $\bar{m}$ is the linear density, and L is the length of the defect (a). The $E_{d}$ is obtained from the relationship between the velocity ($V_{P}$) of the P wave, density (ρ), and Poisson’s ratio (ν) according to Equation (8). Here, for $V_{P}$ of 4580 m/s, ρ of $2400\;\mathrm{kg}/m^{3}$, and ν of 0.2, the dynamic modulus of 45.3 GPa was calculated. The natural frequencies calculated using a series of processes are summarized in Table 2.

(8) $$V_{P}=\sqrt{\frac{E_{d}\left(1-\nu \right)}{\rho \left(1+\nu \right)\left(1-2\nu \right)}}$$

**Figure 13:**
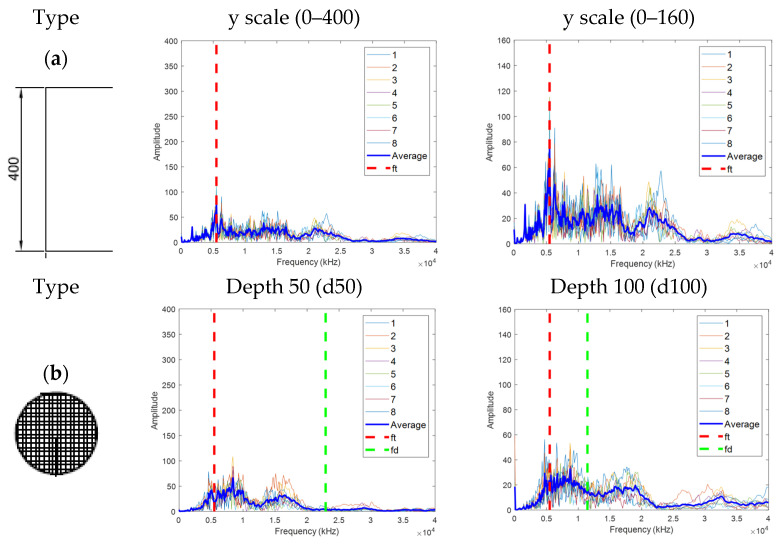

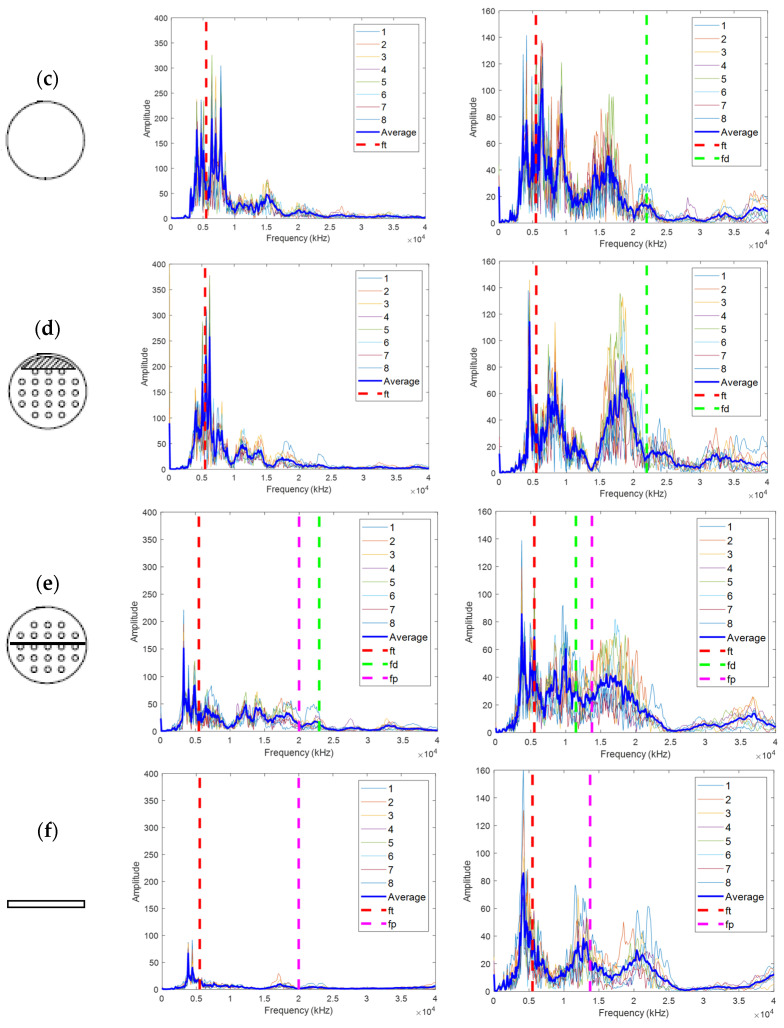
Spectrum results by type for depths of 50 mm (d50) and 100 mm (d100) by IE test. (**a**) N400. Slabs: (**b**) A; (**c**) B; (**d**) C; (**e**) D; (**f**) E.

The *a/d* of A50 ranges from 1.13 to 2.60 depending on the structural shape, and as in the natural frequency calculated in Table 2, strong flexural mode, weak flexural mode, and IE mode may coexist in the range of 2954–10,305 Hz. The *a/d* of B50 ranges from 1.29 to 1.80, and a strong flexural mode appears initially in the natural frequency range of 6164–10,211 Hz, and a weak flexural mode and IE mode may coexist. In addition, *ff* is insignificant or absent, and the IE mode dominates. Because the shape of the upper part of the duct is arcuate rather than flat, the corresponding range was set, and it was verified that strong frequencies within the range occurred through the IE test results in Figure 13.

Figure 13 shows the FFT results for the eight sensors collected through the IE test, and the theoretical values for the possible *ft*, *fd*, and plate defect (*fp*) for each type are shown. In the case of N400, *ft* dominated at 5500 Hz, and the remaining features were negligible. In the case of slab A, it was difficult to transmit energy due to various reflected waves in the arch shape of the duct and the interface between the duct and the filled concrete; therefore, no clear characteristics were observed. In addition, compared with the theoretical value, *ft* and *fd* were shifted to a lower frequency owing to an increase in the movement distance due to the influence of the duct.

Among d50, in the case of B50 and C50, *a/d* > 1.5, *ff* was dominant, and it was difficult to distinguish it from d100, d150, and d200 theoretically. Therefore, d50 should be evaluated mainly based on the characteristics of *ff*. In the case of d100 and d150, as *a/d* < 1.5, *ft* moved to a low frequency, and *fd* and *fp* occurred around the theoretical frequency according to the circumference of the duct or the depth of the acrylic plate. However, as the location of ducts and defects deepened, the reflected energy decreased and the characteristics of each type became weaker, hindering the determination of the theoretical characteristics of d200. In addition, because of the characteristics of the specimen, the characteristics were weakened by the influence of various reflected waves from the surrounding duct, acrylic, and wall. For the three types (A, B, C), the thickness modes *ft* were 4700, 4100, and 4500 Hz, respectively, and the degree of shift of the *ft* was reduced according to the completeness of the grouting. The *ft* of types D and F were 3700 and 4200 Hz, respectively, and the *ft* was shifted to a lower frequency if there was a duct. Although some types of defects could be roughly classified by the occurrence of *ff* and shift of *ft*, this was limited to specific cases. Because there are many factors to consider, such as test conditions, pouring conditions, and concrete properties, it seems difficult to quantify the defects characteristics. 

In this study, the characteristics that could be distinguished by type were analyzed by analyzing the FFT, but there was a limit to distinguishing them based only on the occurrence of peaks and shifts. Therefore, a method for classifying types by extracting various features that maximize the physical properties from the IE spectrum was applied.

### 4.5. Analysis of Defect Types with PCA

#### 4.5.1. Feature Extraction

**Figure 14:**
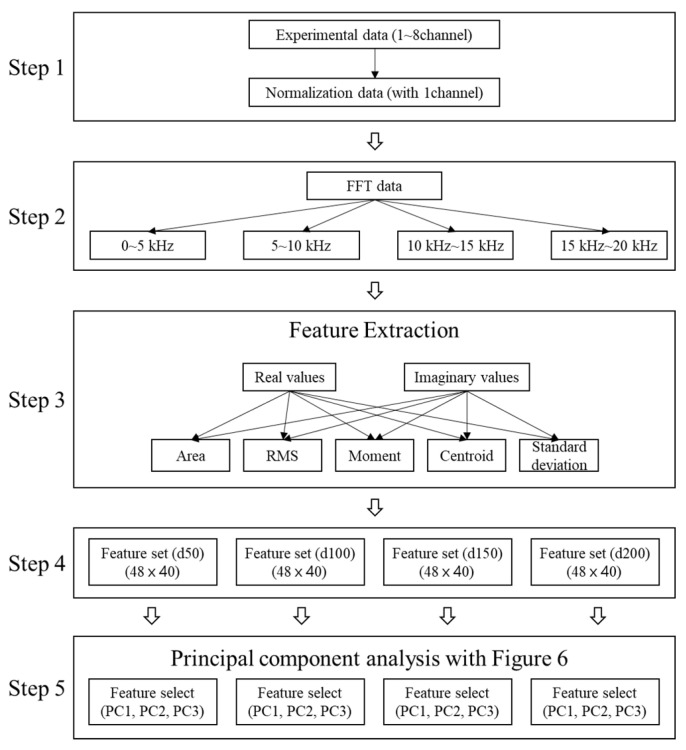
Feature extraction procedure for PCA analysis.

The existing IE spectrum analysis simply detects the occurrence of a new peak in the *ft* shift, energy attenuation, and high frequency based on Equations (1)–(6) for the concrete slab. However, this may be difficult to determine using the theoretical formula in case of changes of the physical properties of concrete or if there are factors that interfere with the propagation of elastic waves such as ducts. In the case of shallow defects, high-frequency generation due to impact is limited; thus, in addition to the analysis of the peak frequency of the spectrum, various other features need to be considered. Moreover, although analysis of ambiguous results may require professional ability to distinguish between defects and non-defects, PCA analysis enables quick and easy evaluations by classifying the distribution ranges of defects and non-defects. Therefore, in the dynamic response according to the type and depth of the duct, the feature that maximizes the classification was extracted by considering *ft* (~5500 kHz), *ff* (2–10 kHz), and *fd* (10–20 kHz). A total of 40 features were extracted through the four-step procedure shown in Figure 14, and the extracted features were used for PCA in the five-step.

#### 4.5.2. Classification Visualization with Principal Components

**Figure 15:**
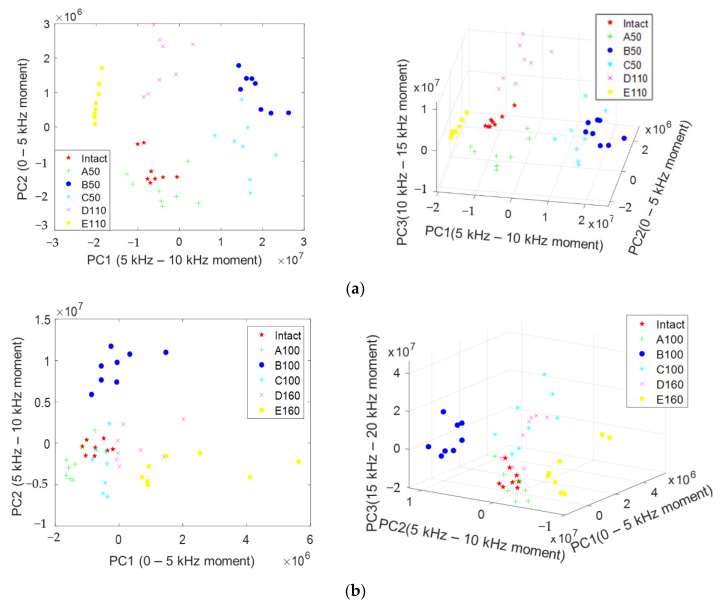

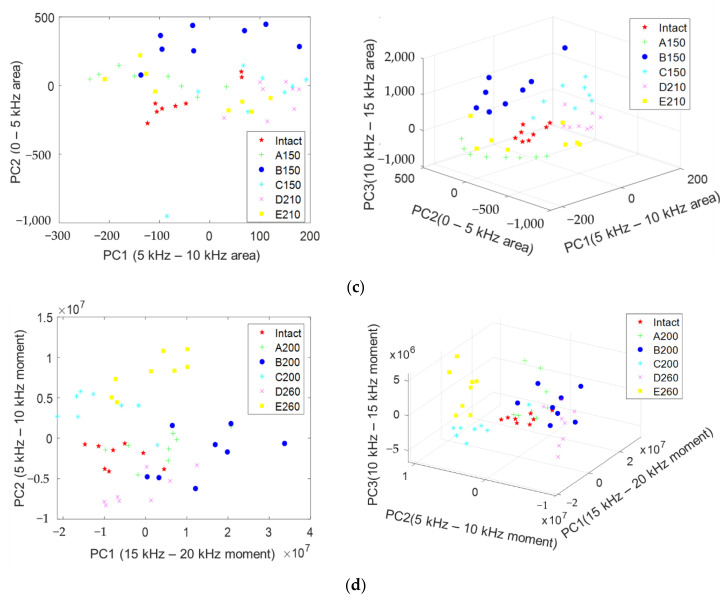
PCA results for defect type and depth. (**a**) Feature set (d50). PC1: 5–10 kHz moment, PC2: 0–5 kHz moment, PC3: 10–15 kHz moment. (**b**) Feature set (d100). PC1: 0–5 kHz moment, PC2: 5–10 kHz moment, PC3: 15–20 kHz moment. (**c**) Feature set (d150). PC1: 5–10 kHz area, PC2: 0–5 kHz area, PC3: 10–15 kHz area. (**d**) Feature set (d200). PC1: 15–20 kHz moment, PC2: 5–10 kHz moment, PC3: 10–15 kHz moment.

Although the depth for a specific type can be estimated through IE, there is a limit to distinguishing them together because intersecting parts occur in the spectra of various depths and defect types. Figure 15 is proposed to accurately determine the depth of the steel duct using the EM waves in Figure 7 and visualize six types of defects according to four depths using two or three principal components (PCs) extracted through PCA. The approach combines the advantages of the two NDT methods and can be effective in actual testing.

Figure 15a shows the results of using the principal components PC1 (5 kHz–10 kHz moment) and PC2 (0–5 kHz moment) for d50. If two PCs are used, 94% of the characteristics are retained; if PC3 is added, up to 99% of the characteristics are retained. The variances of PC1 and PC2 are $2.13^{14}$ and $7.77^{13}$ in the new latent variable space. The intact N400, which is a duct- and defect-free range, and A50 with a duct completely grouted were distributed around it. In the case of A50, as most of the characteristics disappear, it is difficult to distinguish with only the spectrum, but it can be classified as normal through PCA. B50 and C50 are similar defects, and the *ff* mode appears strongly and is distributed on the right side of the graph. D110 and E110 contain similar delamination defects, and although differences occur depending on the presence or absence of ducts, they have similar values in the PC2 standard and are distributed around them. The graph visualizes the values obtained by normalizing the extracted features with PCA. The range in which data are distributed along PC1 and PC2 (x-axis, y-axis) can be considered to be proportional to the energy amplitude of a frequency occurring in the corresponding frequency range. It is possible to overcome the limitations of IE spectrum analysis by analyzing the distribution range of the PCA graph. The d50 type can accurately classify normal, upper, intermediate, and peeling defects using two PCs. In addition, it can be confirmed that they are grouped into similar types. In d100, *a/d* is 1.5 or less; thus, the *ff* mode is weakened and the IE mode is dominant, and most of the spectra amplitudes are similar. Therefore, appropriate feature extraction is required for the peak shifted by type for classification. In Figure 15b, when PC1 (0–5 kHz moment) and PC2 (5–10 kHz moment) were used, B100 and E160 were clearly distinguished according to the degree of shift and weak *ff* of the main mode according to the defect type. The *a/d* of B100 is 1.3, which is a region where weak *ff* and IE modes exist similarly, and it seems that the feature of *ff* occurring above 7600 Hz is well captured. Because the remaining types have overlapping ranges, it was possible to distinguish them through three-dimensional analysis using PC3 (15–20 kHz moment), which is the section where reflected waves from ducts and defects occur. Although some types could be clearly distinguished in d150 and d200, it was difficult to distinguish all six types. This means that as the depth increases, the energy decreases, and it is difficult to distinguish features because of the energy scattering due to the shape of the duct. However, in the PSC structure, because the position of the duct can be identified through EM waves in advance, defects can be sufficiently distinguished by dividing them into a position with a duct (A, C, and D) and a position without a duct (N400, E). In the actual PSC structure, as the case of B (empty duct) is rare, it was excluded; three types of duct interiors, namely, A (fully grouted), C (upper defect), and D (middle defect) could be distinguished; and PCA is expected to be applicable.

**Figure 16:**
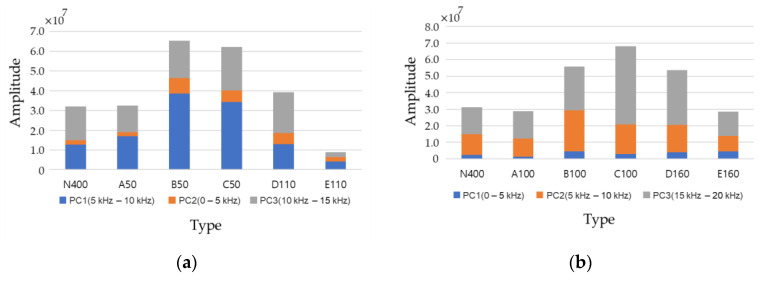

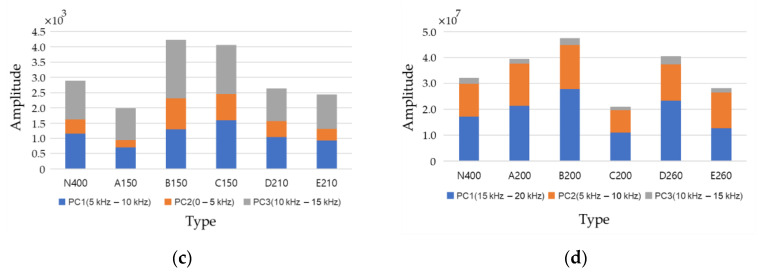
Average of eight sensors for the extracted features. (**a**) d50, (**b**) d100, (**c**) d150, (**d**) d200.

Figure 16 shows a graph of the average values of eight accelerometers in which PCA was not performed for the major variables. Because the average value was used, the size of the variance could not be confirmed, but according to the depth, six types could be distinguished through the ratio of each feature and the sum of three. This analysis shows the possibility of distinguishing six types of states using feature extraction alone.

### 4.6. Integrity Detection Procedure of PSC Structure

**Figure 17:**
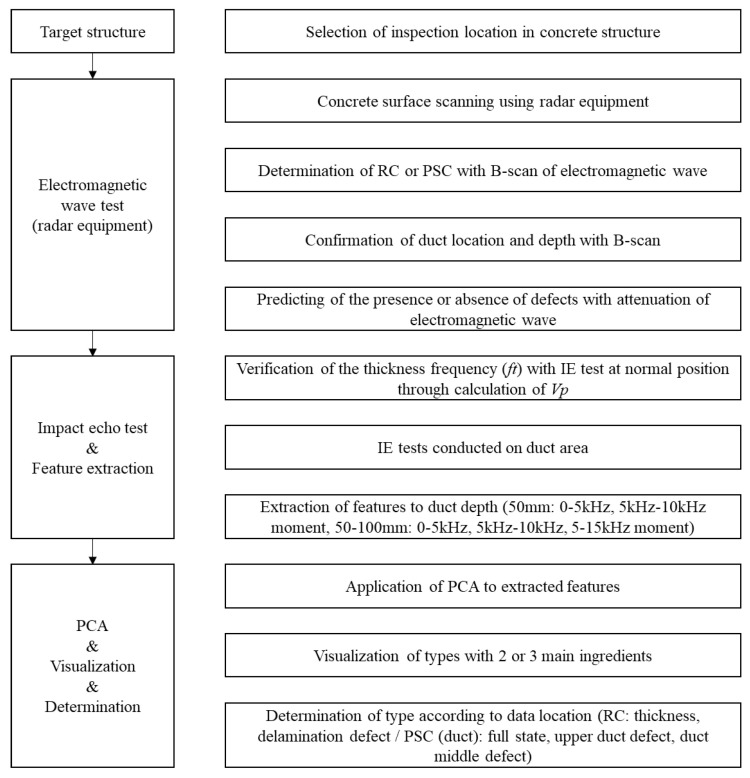
Defect type detection procedure of PSC structure using EM wave and IE.

It is possible to locate a duct with a depth of 50 mm with IE, and a depth of 100 mm can be distinguished from normal and upper defects. However, as the depth increases, the energy scattered by the duct, which is an arcuate structure, increases; therefore, it is difficult to accurately quantify the characteristics. A more advanced method is needed to detect both the type and depth of duct defects using the IE method. When EM waves are used, the position and depth of the duct can be easily detected with a strong reflected wave owing to the difference in permittivity. Therefore, in this study, a method for estimating the internal shape and defects by first detecting the position and depth of the duct with respect to the PSC structure using EM waves, and then identifying the characteristics of each stage by IE, is proposed, as shown in Figure 17. It is expected that the proposed procedure can be used more effectively if RC and PSC are applied separately.

## 5. Conclusions

In the PSC structure, which is widely used in modern bridge construction, the evaluation of the condition of the tendon duct is important. Tendon corrosion and delamination defects caused by insufficient grouting reduce the reliability and durability of structures. Previously, EM waves were used to locate steel ducts, and puncture by sampling and drilling was performed to evaluate the lack of grout. The IE method applied thereafter simply evaluated the state through the occurrence of a theoretical peak in the spectrum, but this method was difficult to be applied for state evaluation because the degree of shift of the frequency depending on the conditions was not clear. In this study, EM waves, S waves, IE test, and MASW were applied for state evaluation of PSC structures, and the following conclusions were drawn by analyzing the limitations and possibilities of each method:

- Although it was difficult to identify defects inside the duct through the general B-scan of the RC radar, they could be roughly inferred from the energy attenuation degree of energy through the Hilbert transformation of the extracted raw data.
- Array equipment using S waves showed similar B-scan results with voids, tendons, and delamination defects; thus, there is a limit to evaluate the defects of the PSC structure.
- In the MASW, some features of the A0 and S1 modes were identified for delamination defects described with normal N400 and acrylic plates. However, it is difficult to apply the MASW analysis based on an infinite plate, because most of the features disappear in the arched duct and do not follow the theoretical curve.
- In the IE test, when the inside of the duct was completely grouted (A), it was difficult to transmit energy because of the reflection of the duct and the interface between the duct and the filled concrete; thus, no clear features were identified other than the thickness mode (*ft*).
- In the IE spectra of A, B, C, and D with N400 and duct, *ft* was in the order of A > C > B > D, and there was a difference in the shift depending on the degree of grouting and the depth of the defect. The largest shift occurred in the intermediate defect (D), which is considered to be because the elastic wave propagating into the duct was shifted once more through the middle delamination defect, and the movement distance increased. However, this seems difficult to quantify, as the spectrum may change depending on the test conditions and physical properties of concrete.
- Because it is difficult to consider all the effects of the duct through the occurrence and change of frequency in the general IE spectrum, in this study, features such as moment and area were extracted by considering the frequency range where theoretical *ft*, *ff*, and *fd* occur for each defect type. This was effective in classifying the six types and might be used for the activation and development of the IE method.
- In the case of d150 and d200, the energy gradually weakened depending on the impact position and positions 1–8 of the sensor; thus, an overlapping section occurred in the spectrum, limiting the application of the six types. However, if the position and depth of the duct could be determined using EM waves, it would be sufficiently applicable to detect fully grouted conditions, lack of grouting, and delamination defects in ducts.
- In this study, we proposed a procedure for specifying the location and burial depth of steel ducts using EM waves and classifying defect types through IE and PCA. The proposed procedure seems to be applicable to bridge deck slabs and can contribute to the convergence of various technologies in IE.

## Figures and Tables

**Table 1:** Summary of the three theoretically possible modes and *a/d* results.

Type	*a/d*	*ft*	*ff*	*fd*	Type	*a/d*	*ft*	*ff*	*fd*
N400	-	o	x	x	-	-	-	-	-
A50	-	o	x	o	A150	-	o	x	o
B50	2.60	x	o	o	B150	0.87	o (shift)	x	o
C50	1.80	x	o	o (shift)	C150	0.60	o (shift)	x	o (shift)
D50	1.13	o (shift)	x	o (shift)	D150	0.60	o (shift)	x	o (shift)
E50	1.13	o (shift)	x	o	E150	0.60	o (shift)	x	o
A100	-	o	x	o	A200	-	o	x	o
B100	1.30	o (shift)	x	o	B200	0.65	o (shift)	x	o
C100	0.90	o (shift)	x	o (shift)	C200	0.45	o (shift)	x	o (shift)
D100	0.79	o (shift)	x	o (shift)	D200	0.49	o (shift)	x	o (shift)
E100	0.79	o (shift)	x	o	E200	0.49	o (shift)	x	o

**Table 2:** Summary of information for natural frequency calculations.

Type	*n*	*a (m)*	*d (m)*	$\omega_{n}\;(\mathrm{Rad}/s)$	Natural Frequency (Hz)	*a*/*d*
A50 (top)	1	0.13	0.05	$1.856^{4}$	2954	2.60
A50 (end)	1	0.13	0.115	$6.475^{4}$	10,305	1.13
B50 (top)	1	0.09	0.05	$3.873^{4}$	6164	1.80
B50 (end)	1	0.09	0.07	$6.416^{4}$	10,211	1.29

## Data Availability

The data presented in this study are available upon request from the corresponding author.

## References

[1] Mathy B., Demars P., Roisin F., Wouters M. Investigation and strengthening study of twenty damaged bridges: A Belgium case history. Proceedings of the Third International Conference on Bridge Management.

[2] Youn S., Kim E. Deterioration of bonded posttensioned concrete bridges and research topics on the strength evaluation in ISARC. Proceedings of the JSCE–KSCE Joint Seminar on Maintenance and Management Strategy of Infrastructure in Japan and Korea, JSCE Concrete Committee Newsletter.

[3] Concrete Society (2006). Durable Bonded Post-Tensioned Concrete Bridges.

[4] Woodward R.J., Williams F.W. (1988). Collapse of YNYS-Y-GWAS bridge, Glamorgan. Proc. Inst. Civ. Eng..

[5] Woodward D.J., Wilson D.L.S. (1991). Deformation of segmental post-tensioned precast bridges as a result of corrosion of the tendons. ICE Proc..

[6] Chen W.F., Duan L., Place H. (2014). Handbook of International Bridge Engineering.

[7] Gucunski N., Basily B., Kim J., Duong T., Maher A., Dinh K., Azari H., Ghasemi H. Assessing condition of concrete bridge decks by robotic platform RABIT for development of deterioration and predictive models. Proceedings of the 8th International Conference on Bridge Maintenance, Safety and Management (IABMAS).

[8] Reichling K., Raupach M., Wiggenhauser H., Stoppel M., Dobmann G., Kurz J. BETOSCAN—Robot controlled non-destructive diagnosis of reinforced concrete decks. Proceedings of the NDTCE’09, NonDestructive Testing in Civil Engineering.

[9] Krause M., Milmann B., Mayer K., Schickert M. Investigation of Tendon Ducts by Means of Ultrasonic Echo Methods: A Comparative Study. Proceedings of the 9th European Conference on NDT (ECNDT 2006).

[10] Dawood N., Marzouk H., Hussein A., Gillis N. (2013). Nondestructive assessment of a jetty bridge structure using Impact-Echo and Shear-Wave techniques. ASCE.

[11] Sansalone M.J., Streett W.B. (1997). Impact-Echo: Nondestructive Evaluation of Concrete and Masonry.

[12] Sansalone M.J. (1997). Impact-Echo: The complete story. ACI Struct. J..

[13] Grosse C.U., Reinhardt H.W., Krüger M., Beutel R. Application of Impact-Echo techniques for crack detection and crack parameter estimation in concrete. Proceedings of the 11th International Conference on Fracture (ICF11).

[14] Sansalone M.J., Carino N.J. (1989). Detecting delaminations in concrete slabs with and without overlays using the impact-echo method. ACI Mater. J..

[15] Carino N.J., Sansalone M., Nowak A.S. (1990). Flaw Detection in Concrete Using the Impact-Echo Method. Bridge Evaluation, Repair and Rehabilitation.

[16] Garbacz A., Piotrowski T., Kwasniewski L., Courard L. (2017). On the evaluation of interface quality in concrete repair system by means of impact-echo signal analysis. Constr. Build. Mater..

[17] Schubert F., Köhler B. (2008). Ten Lectures on Impact-Echo. J. Nondestruct. Eval..

[18] Xiao J.Z., Agrawal A. (2015). Robotic Inspection of Bridges Using Impact-Echo Technology.

[19] Krüger M., Grosse C.U. (2007). Impact-Echo-Techniques for crack depth measurement. Sustain. Bridges.

[20] Chaudhary M.T.A. (2013). Effectiveness of impact echo testing in detecting flaws in prestressed concrete slabs. Construct. Build. Mater..

[21] Liang M.T., Su P.J. (2001). Detection of the corrosion damage of rebar in concrete using impact-echo method. Cem. Concr. Res..

[22] Song K.I., Cho G.C. (2009). Bonding state evaluation of tunnel shotcrete applied onto hard rocks using the impact-echo method. NDT E Int..

[23] Valenzuela M.L., Sansalone M.J., Streett W.B., Krumhansl C.L. Use of sound for the interpretation of impact-echo signals. Proceedings of the 4th International Conference on Auditory Display (ICAD1997).

[24] Carino N.J., Sansalone M.J., Hsu N.N. (1986). A point sourcepoint receiver, pulse-echo technique for flaw detection in concrete. ACI J..

[25] Sansalone M., Carino N.J. (1986). Impact-Echo: A Method for Flaw Detection in Concrete Using Transient Stress Waves.

[26] Lee Y.H., Oh T.K. (2016). The simple lamb wave analysis to characterize concrete wide beams by the practical MASW test. Materials.

[27] Song H.M., Hong J.Y., Choi H.J., Min J.Y. (2021). Concrete delamination depth estimation using a noncontact MEMS ultrasonic sensor array and an optimization approach. Appl. Sci..

[28] Chen H., Xu B., Wang J., Luan L., Zhou T., Nie X., Mo Y.L. (2019). Interfacial debonding detection for rectangular CFST using the MASW method and its physical mechanism analysis at the Meso-Level. Sensors.

[29] Abraham O., Cote P. (2002). Impact Echo Thickness Frequency Profiles for detection of voids in tendon ducts. ACI Struct. J..

[30] Liu Y.L., Shi J.J., Huang J.Q., Wei G.S., Wu Z.X. (2019). Grouting defect detection of lapped bar connections based on Impact-Echo method. Shock. Vib..

[31] Tinkey Y., Olson L.D. (2007). Impact-Echo Scanning for Grout Void Detection in Post-tensioned Bridge Ducts-Findings from a Research Project and a Case History. ASCE.

[32] Zou C., Chen Z., Dong P., Chen C. (2016). Experimental and numerical studies on nondestructive evaluation of grout quality in tendon ducts using Impact-Echo method. ASCE.

[33] Zhang J.K., Yan W., Cui D.M. (2016). Concrete condition assessment using Impact-Echo method and extreme learning machines. Sensors.

[34] Oh B.D., Choi H., Song H.J., Kim J.D., Park C.Y., Kim Y.S. (2020). Detection of defect inside duct using recurrent neural networks. Sens. Mater..

[35] Oh T.K., Park J.I., Byun Y.S., Lee Y.H. (2015). A study on the influence factors on flexural and thickness modes in the Impact-Echo test. J. Comput. Struct. Eng. Inst. Korea.

[36] Hotelling H. (1993). Analysis of a complex of statistical variables into principal components. J. Educ. Psychol..

[37] Jollie I.T. (2002). Principal Component Analysis.

[38] Choi I.H., Son J.A., Koo J.B., Yoon Y.G., Oh T.K. (2019). Damage assessment of porcelain insulators through principal component analysis associated with frequency response signals. Appl. Sci..

[39] Rao S.S. (2007). Vibration of Continuous Systems.

